# Geopsychiatry and the complex mental health challenges of conflict: a review examining local and distal psychiatric outcomes in a fractured world

**DOI:** 10.1007/s40211-025-00567-x

**Published:** 2026-01-08

**Authors:** Alexander Smith, Juan Graña, Rowalt Alibudbud, Albert Persaud, Julio Torales, Ana Buadze, Michael Liebrenz

**Affiliations:** 1 Department of Forensic Psychiatry, University of Bern, Hochschulstrasse 4, 3012 Bern, Switzerland; 2 Department of Psychiatry and Behavioral Medicine, University of the Philippines—Philippine General Hospital, Manila, Philippines; 3 Institute of Psychiatry, Psychology & Neuroscience, King’s College London, London, UK; 4 Facultad de Ciencias Médicas, Grupo de Investigación sore Epidemiología de los Trastornos Mentales, Psicopatología y Neurociencias, Universidad Nacional de Asunción, San Lorenzo, Paraguay; 5 Department of Psychiatry, Psychotherapy and Psychosomatics, Psychiatric Hospital, University of Zurich, Zurich, Switzerland; 6 Department of Sociology and Behavioral Sciences, De La Salle University, Manila, Philippines; 7 Vicerrectoría de Investigación y Postgrado, Universidad de Los Lagos, Osorno, Chile; 8 Facultad de Ciencias de la Salud, Universidad Sudamericana, Pedro Juan Caballero, Paraguay

**Keywords:** Geopsychiatry, Conflict, Mental Health, Geopolitical determinants, Vulnerable populations, Geopsychiatrie, Konflikt, Psychische Gesundheit, Geopolitische Einflussfaktoren, Schutzbedürftige Bevölkerungsgruppen

## Abstract

Armed conflicts can engender substantial detrimental effects for mental health, both within immediate settings and for faraway populations. Guided by frameworks from geopsychiatry, this narrative review underlines the local- and macro-level psychiatric consequences of conflicts, informed by illustrative findings from academic sources and gray literature focusing on contemporary global disputes. Specifically, the review demonstrates that exposure to conflict is consistently associated with elevated rates of mental illnesses, which are worsened by devastated healthcare systems and related phenomena. Subsequently, it highlights how vulnerable populations often experience disproportionate mental health burdens in conflict, before exploring how secondary, distal psychiatric effects can arise from vicarious media exposure, forced displacement, and associated environmental degradation. With the most armed hostilities occurring since the end of World War Two, the paper concludes by considering how the psychiatric discipline can adapt to confront the modern challenges of diversifying patient populations in an increasingly turbulent geopolitical landscape.

## Abstract (de)

Bewaffnete Konflikte können substanzielle schädliche Effekte auf die psychische Gesundheit mit sich bringen, sowohl in der unmittelbaren Umgebung als auch bei weit entfernt befindlichen Populationen. Anhand von Rahmenbedingungen aus der Geopsychiatrie werden in der vorliegenden narrativen Übersicht auf lokaler und Makroebene die psychiatrischen Folgen von Konflikten mittels anschaulicher Erkenntnisse aus wissenschaftlichen Quellen und grauer Literatur mit Fokus auf zeitgenössische weltweite Diskussionen verdeutlicht. Insbesondere zeigt die Übersicht, dass die Exposition gegenüber Konflikten in konsistenter Weise mit erhöhten Raten psychischer Erkrankungen einhergeht, die noch durch vernichtete Gesundheitssysteme und damit zusammenhängende Phänomene verschlimmert werden. In der Folge wird hervorgehoben, wie vulnerable Populationen oft unter unverhältnismäßigen psychischen Lasten in Konflikten leiden, bevor dargelegt wird, wie sekundäre, distale psychische Effekte aus der Exposition gegenüber indirekter Medienpräsenz, gewaltsamer Vertreibung und assoziierter Umweltzerstörung entstehen können. Während zurzeit die meisten bewaffneten Auseinandersetzungen seit Ende des Zweiten Weltkriegs stattfinden, endet die Arbeit mit der Betrachtung, wie das psychiatrische Fachgebiet sich anpassen kann, um die modernen Herausforderungen immer mannigfaltigerer Patientenpopulationen in einer zunehmend turbulenten geopolitischen Landschaft zu bewältigen.

## Introduction

We live in a tumultuous epoch, shaped by digital advancements and medical innovations, yet shadowed by geopolitical frictions and societal uncertainty [1]; it may be the best of times, it may be the worst of times. In today’s interdependent and globalized systems, large-scale events and circumstances can have far-reaching local repercussions, influencing the contours of everyday existence [1–5]. Mental health is no exception and is especially sensitive to exogenous forces. Underpinned by a range of biopsychosocial factors, macro-level determinants can impinge upon (and are already affecting) the well-being and living situations of individuals, communities, and populations [2–5].

This is exemplified by data revealing the associations between planetary climate change and psychiatric symptoms [3, 4, 6]. Likewise, mass migratory movements can amplify pre-existing pressures, both personal and systemic, potentially engendering distinctive stressors for those who move to a different country and their host areas [3–5, 7]. Notwithstanding its possible benefits, the psychosocial implications of job losses and techno-insecurity resulting from artificial intelligence (AI) “productivity gains” have also not been fully scrutinized [8].

In response to this, the subdiscipline of geopsychiatry seeks to interrogate and elucidate how macro-level geopolitical determinants can cascade into proximal mental health outcomes [2–5]. In tracing these linkages, geopsychiatry promotes integrated approaches for understanding how wider instabilities manifest as psychiatric vulnerabilities. Concomitantly, epistemological models in geopsychiatry drawn upon clinical, sociocultural, and sociopolitical knowledge exchanges that transcend borders, but are generally informed by (and predominantly originate from) regions characterized by geopolitical crises [2–5].

In this context, inter- and intra-state disputes and other conflicts have become a core focus for geopsychiatry, given their multifaceted foundations, coupled with their propensity to cause immediate and enduring psychiatric harms [5, 9, 10]. Specifically, exposure to armed combat and mass violence can precipitate and worsen psychiatric risk factors and symptoms, often disproportionately among predisposed subpopulations, while provoking longer-term mental health issues [5, 9–12]. Concurrently, psychiatric care services can collapse in conflict zones, disrupting essential resource provisions and degrading mental health delivery [5, 9–12]. More broadly, conflicts can crystallize and exacerbate adverse structural conditions and phenomena, all of which may conceivably translate into downstream psychiatric effects [5].

Since 1800, over 37 million people have died fighting in wars worldwide, a number far higher when incorporating civilian deaths [13]. Today, violence is surging to levels unseen since World War Two. In 2024, 61 conflicts spanned 36 countries throughout Africa, Europe, the Middle East, South America, and elsewhere, with many states experiencing multiple wars at once [14]. Moreover, conflicts entail immense socioeconomic damage; in 2023, global violence cost an estimated $19.1 trillion (13.5% of the world’s gross domestic product [14]). These realities reinforce the need for greater sensitivity toward the impact of armed combat on the development, distribution, and intensification of mental health concerns within conflict zones themselves and across larger transnational spheres [9]. In turn, psychiatric care pathways and training schemes will likely need to adapt to these urgent challenges [5, 11].

Thus, in line with concepts in geopsychiatry, this narrative review examines these intersecting dimensions, supplemented by academic sources and gray literature from recent conflicts. To that end, it first surveys general patterns in war- and conflict-related psychiatric outcomes, then accentuates the differential consequences for marginalized and vulnerable groups through illustrative examples. Subsequently, it outlines secondary (but psychiatrically relevant) correlates of conflict, and concludes with reflections on the changing responsibilities of mental health specialists in managing the professional difficulties of a turbulent international order.

## The psychiatric consequences of war and armed conflict

Conflict-related psychiatric morbidities are both a central concern for geopsychiatry and a major public health issue [11, 12]. Reliably assessing mental health in conflict zones is challenging (and, at times, implausible); insecurities inevitably hamper epidemiological data collection, as can military and governmental sensitivities [11, 12, 15]. Still, a substantial body of work emphasizes how armed disputes trigger the onset of psychiatric conditions, worsen pre-existing mental illnesses, and heighten the incidence of critical life events.

Notably, a meta-analysis spanning multiple conflict-afflicted areas determined that 22.1% of people in these environments experienced some form of mental disorder [12]. The prevalence of certain mental illnesses in conflict-exposed individuals has repeatedly been found to be higher than normative baselines, with one study identifying a two- to threefold increase in anxiety, depression, and posttraumatic stress disorder (PTSD [11, 16]). Alongside non-combatants, veteran subpopulations face a heavy mental burden [17]. Increasingly, research has also explored the potential for the intergenerational transmission of trauma, especially in settings where mass violence and atrocities are sustained over prolonged periods [9, 18].

However, diagnostic changes render like-for-like comparisons across conflicts imperfect (if not delimiting); indeed, nosological criteria and terminology, especially for classifying trauma, have shifted substantially over generations [19]. Yet, the symptomatology recorded under earlier labels (i.e., “shell shock,” “combat fatigue,” and “traumatic neurosis”) closely map onto what is now captured by PTSD and related diagnoses [19]. Prevalence estimates should therefore be interpreted with caution, privileging symptom-level and functional indicators where possible.

That notwithstanding, regional findings illustrate the scale of extant psychiatric needs. For example, the World Health Organization suggested that up to 10 million Ukrainians were at risk of developing psychiatric disorders months after the full-scale Russian invasion of Ukraine [20]. Subsequently, as this war has escalated, so too have psychiatric hospitalizations [21]. Meanwhile, persistent violence in the Palestinian Territories has coincided with a sizeable occurrence of mental illnesses and ongoing complex trauma [22, 23].

During Sudan’s civil war, 56.9% of survey respondents in this jurisdiction had probable PTSD, rising to 60.5% for frontline Sudanese healthcare workers [24]. Elsewhere, in Kashmir, one of the most heavily militarized areas in the world, 45% of surveyed adults presented with mental distress [25]. Moreover, for armed personnel, rates of substance use and other psychiatric disorders can be significant; this was apparent among members of the United States military in the wars in Afghanistan and Iraq, together with individuals in different national forces [17, 26].

Within wider conflict-affected populations, mental illnesses may co-occur with somatic issues and infectious diseases (e.g., blast-related injuries, traumatic brain injury, tuberculosis, or HIV/AIDS), as well as being influenced by distinctive psychosocial stressors (e.g., ongoing threats of harm, socioeconomic issues, and bereavement [9–11, 14–16]). In Haiti, sociopolitical instability, gang violence, and humanitarian collapse have engendered pervasive “chronic traumatic stress” [27]. In the Tigray War in Ethiopia, cultural and financial losses have intensified psychological challenges and undermined community resilience [28]. Additionally, research from Africa, Asia, Europe, and the Middle East has elucidated the links between human rights abuses and detrimental mental health outcomes [29].

Equally, external and internal displacement, food shortages, livestock destruction, and malnutrition are well-established psychiatric risk factors in times of conflict [5, 9–12, 22, 23, 28, 30]. By the end of 2024, approximately 123 million people were forcibly displaced globally, rising on prior years, with the continuing intra-state conflict in Sudan producing unprecedented levels of displacement and hunger [31, 32]. These dynamics have been observed in numerous other conflict-affected regions including (but not limited to) the Democratic Republic of Congo, Myanmar, Nagorno-Karabakh, the Palestinian Territories, Ukraine, and Venezuela [22, 23, 34–36].

Widespread disruption to healthcare services compounds these scenarios of disadvantage, and can be accelerated by resource deficiencies and indiscriminate infrastructural attacks [9–12, 22, 28]. By 2021, roughly half of Syria’s medical facilities were defunct following the civil war that commenced in 2011 [37]. Analogously, in Yemen, years of armed combat have desolated many hospitals and clinics [38], while psychiatric facilities have been bombarded or occupied by Russian forces in Ukrainian cities [39].

Indeed, healthcare services are frequently destroyed (and often deliberately targeted, in breach of international law), with pharmaceutical supply chains also impeded [9–12, 22–24, 27, 36, 40–42]. Despite refinements to psychiatric interventions in conflict zones, most affected populations lack access to adequate psychosocial support and treatment [30, 43, 44]. Similarly, in the longer term, civilian and veteran groups alike routinely encounter psychiatric care barriers, aggravated by insufficient funding and infrastructure [9–12, 17].

Further constraints arise from the emigration of highly skilled health professionals from conflict-affected regions. In protracted crises, physicians and trainees may flee early, magnifying treatment gaps and eroding supervisory and training capacity. Again, Syria offers a stark illustration, with ~70% of its healthcare workforce escaping during its civil war [36]. These shortages can be exacerbated by general patterns of “push” and “pull” factors amplifying outward physician migration from low-to-middle-income countries [45].

## The disproportionate impact of war and conflict on vulnerable groups

Although widely felt, conflict-related psychiatric sequalae can excessively impact marginalized and vulnerable groups [5, 9–12, 46]. As the ensuing examples outline, this includes children and adolescents, women, older adults, socioeconomically disadvantaged groups, people living with disabilities, and individuals in detention facilities, among others [5, 9–12, 46].

Notably, at the time of writing, around 473 million children (~19% of the global child population) live in conflict-afflicted regions, where they can be exposed to extreme violence, displacement, malnutrition, and the loss of family members [46–50]. Educational pathways are repeatedly disrupted, and in certain contexts, armed factions have coercively enlisted children and adolescents [46–50]. These factors can all profoundly impair developmental trajectories and functioning, contributing to immediate and longer-term behavioral and psychiatric challenges [46–50].

International data underline these trends. In a sample of displaced school-age children in the Sudanese civil war, 29% had diagnosed mental health disorders and the majority of participants had sub-pathological psychological issues [51]. In Colombia, internally displaced adolescents exhibited a substantial mental health burden compared to non-displaced peers, particularly for PTSD (12.3% vs. 2.1%) and suicidal ideation (19.8% vs. 5.8% [52]). Meanwhile, in another study, Ukrainian adolescents in the Russo-Ukrainian War presented with psychiatric symptoms, ranging from 17.9% screening positive for moderate or severe anxiety to 35.0% for clinically relevant trauma [53]. After Myanmar’s 2021 military coup, adolescent girls exhibited sizeable levels of depressive symptoms and exposure to traumatic conflict-related stressors, which were both correlated [54]. Additional results from conflict-affected jurisdictions, such as the Palestinian Territories, the Philippines, Uganda, and Sri Lanka, reveal analogous patterns [20, 46–50, 55].

Throughout different theaters of war, women have been prone to gender-based harms, especially sexual violence, with concurrent mental health effects [11, 46]. This has transpired in the Democratic Republic of Congo, where incidents of rape have risen amid the insurgency of Rwanda-backed M23 forces [56]. In Ethiopia’s Tigray War, 75.6% of women and girls who participated in research around sexual violence had mental health and behavioral problems (e.g., 38.6% with anxiety and 35.2% with sleep disturbances [56]). Complex caregiving demands can further undermine the mental well-being of women and girls caught up in conflicts, as can disruptions to medical care (e.g., maternal health care [57, 58]).

Equally, older adults are susceptible to distinctive psychiatric implications of conflict. Limited mobility and extant health conditions can hinder access to evacuation and essential services [59, 60]; these situations have been documented in conflict zones across the world, such as Venezuela and South Sudan [61]. Relatedly, in various settings, human rights violations and the arbitrary detention of older adults have been highlighted, including egregious accounts of state security forces in Myanmar forcing older people back into burning buildings [59]. Albeit understudied, displacement has significant associations with mental health issues in older men and women, in part influenced by acculturation pressures and critical life events in migratory journeys [60, 61].

Communities in underserved areas also face heightened psychiatric risks from conflict, as seen in the 2017 Marawi Siege in Lanao del Sur, Philippines, where 71.9% of residents already lived below the poverty line [62]. This event displaced thousands of people, triggering a mental health crisis with over 30,000 individuals needing care, but limited professionals and urban-centered services necessitated a reliance on irregular aid or herbal remedies [62, 63]. Likewise, in rural Sierra Leone, decades of civil war and the Ebola outbreak left many communities with high rates of untreated trauma and depression, compounded by insufficient mental health services [64].

Separately, people living with disabilities and serious illnesses face accentuated risks in conflict [65–67]. Akin to other vulnerable groups, Human Rights Watch noted that people living with disabilities endure greater dangers in escaping violence and lack assistive devices, resulting in psychological distress [67]. Finally, individuals in detention and intramural environments (where there are already elevated rates of mental health issues) might encounter psychological harms during episodes of armed combat. Recently, this became apparent through attacks on inpatient hospitals and the systematic conscription of offenders in the Russo-Ukrainian War [39, 68].

## Secondary and macro-level correlates of war and conflict for mental health

As the reviewed literature shows, the psychiatric impact of conflict reaches far beyond groups and communities primarily affected. From the perspective of geopsychiatry, conflict shapes psychiatric morbidity directly and can cascade down through larger sociopolitical, economic, and ecological systems [3–5]. Specifically, conflict-driven psychiatric issues often filter into faraway communities, whether via vicarious psychological repercussions, large-scale displacement, conflict-driven environmental breakdown (which are all explicitly described below), or other secondary correlates.

One example is the way in which viewing media coverage of conflict and associated concerns about war can induce psychological distress in different populations [69]. Although indirect exposure is not a qualifying stressor for PTSD in DSM-5/DSM-5-TR (unless occupationally related), vicarious stressors can still provoke clinically relevant symptoms and functional problems [9]. Compared with earlier eras, modern algorithmically amplified platforms now deliver large volumes of graphic, user-generated war-related content [70]. Concerningly, dose–response studies demonstrate how more exposure to war-related news is associated with higher distress, partly via rumination, and repeated media consumption of collective trauma predicts acute stress responses [71, 72].

Notably, in Portugal, people who were close-tracking the Russo-Ukrainian War exhibited stress levels and PTSD-like symptoms [73]. Similarly, in Italy and Germany, fear of war was correlated with probable anxiety and depression, which is underpinned by comparable findings from other jurisdictions [70, 74, 75]. Consequently, guidance from established organizations now recommends limiting exposure to graphic coverage and setting intentional news boundaries, especially for young people and for those who experienced prior trauma (e.g., [76, 77]).

Displacement further illustrates the complex macro-level and proximal psychiatric implications of conflict. As detailed earlier, conflict-induced displacement and related mental health issues have surged [31, 52, 53, 60, 61]. Concomitantly, the scale of current refugee movements, combined with attendant demands for shelter, resettlement, and integration services, can generate ripple effects outside the original theaters of war. In Jordan, refugee camps pressurized sewage systems and water supplies, and in Lebanon, a rapid influx of people fleeing violence strained already overstretched health, water, hygiene, and sanitation services [78, 79]. Devoid of coordinated service planning and funding, these circumstances can destabilize the living conditions and well-being of both refugees and local populations [80].

In parallel, the capacity of psychiatrists in host countries to meet growing needs is constrained by a demand–supply mismatch and policy environments that restrict access. Refugees typically exhibit elevated rates of psychiatric disorders, yet language, legal precarity, and knowledge gaps hamper care [81]. Correspondingly, in many high-income jurisdictions, anti-migration rhetoric frequently portrays asylum seekers and refugees fleeing violence as competitors for jobs, housing, or social services, and even as public security threats [82, 83].

Often weaponized for political capital, evidence suggests that belief in such perceptions can heighten hostility and anxiety among members of the host community [83]. In the Netherlands, one study identified that the mental health of residents worsened after refugees were resettled in their neighborhood, despite no measurable increase in actual threats [84]. Significantly, within this sample, those already fearful of refugees saw the most deterioration in their mental well-being [84].

Military activities are an underacknowledged but substantial source of greenhouse gas emissions. The world’s armed forces account for around ~5.5% of global carbon output, ranking them among some of the largest emitters when aggregated [85, 86]. Locally, the destruction of agricultural land, livestock, and the release of hazardous substances in conflict-afflicted areas degrades ecosystems and can undermine collective well-being [85, 86]. In parts of East Africa, environmental damage caused by prolonged hostilities has been connected to eco grief and other modalities of psychological distress [87].

More broadly, however, this can have downstream mental health effects. Carbon emissions and ecological ruination may exacerbate climate-related stressors, for example, through heatwaves, floods, droughts, and socioeconomic instability, all of which could conceivably aggravate rates of psychiatric morbidity [3, 4, 6, 88, 89]. Likewise, conflict-induced resource depletion has propagated through food and energy supply chains, elevating prices and insecurity in importing regions with possible contingent psychosocial consequences [3, 4, 6, 85, 86]. Hence, these paradigms may operate as compounding mechanisms, intensifying socioenvironmental pressures that can impinge upon populational mental health; incidentally, the sad irony is that climate change likely escalates the potential for further armed disputes, creating deleterious and self-reinforcing cycles [90].

## Conclusions

This review highlights how wars worsen mental health outcomes across multiple levels and spheres. Confronted with escalating contemporary conflicts, modern psychiatry will need to adapt to these realities [5, 9, 10]. Currently, there is no more pressing time or greater moral duty for practitioners to speak out about safeguarding vulnerable groups [5, 91]. Previously, advocacy schemes involving psychiatrists and professional associations have contributed to curtailing injurious practices, like solitary confinement of offenders with mental illnesses and similar unethical phenomena [5, 92]. Elsewhere, physicians have campaigned for nuclear disarmament and other peace-related causes [93, 94].

Accordingly, individual psychiatrists and representative bodies should reaffirm the importance of interstitial policy changes that can help support mental health in times of conflict and potentially mitigate wider harms; for example, this could entail championing evidence-based practices for psychosocial assistance in conflict zones, adherence to principles of human rights, targeted public mental health strategies, and the consideration of psychiatric factors in humanitarian aid programs [10, 29, 92, 95].

For the latter, the CAPE-VI Index offers policymakers a useful tool for targeting vulnerable countries, allocating aid independently of unreliable partners, and advancing both global health and national interests [96]; such new considerations and innovations may be particularly pertinent amid largescale cuts to overseas aid internationally and a disintegrating global order [96]. Equally, throughout post-conflict settings, psychiatrists have successfully pioneered systemic reforms [5]. Specifically, local practitioners in Bosnia and Herzegovina and Kosovo collaborated to reform and modernize mental health care following the Bosnian and Kosovan wars, informed by experiences from these conflicts [97, 98].

Nevertheless, the reality is that these initiatives, despite their relevance and value, are unlikely to have a significant effect on halting ongoing hostilities and redressing underlying geopolitical determinants. In this sense, alone, the psychiatric discipline’s influence on the overarching courses of conflict can be negligible. Thus, outside of warzones, the most meaningful professional contributions could well lie in pragmatic adaptations to address current and arising issues.

To that end, an important first step would be to ensure that mental health specialists are sufficiently equipped to engage in advocacy, as well as to handle the emerging complexities provoked by armed combat [5, 9, 10, 41, 99]. Presently, conventional training in many regions typically lacks comprehensive coverage of conflict-related psychiatric concerns [10, 41]. With disputes intensifying over numerous regions and credible forecasts pointing to further belligerence in the years ahead, today’s workforce and tomorrow’s trainees risk remaining underprepared to recognize and manage the symptom profiles they will encounter [5, 9, 10, 41, 91, 99].

Concomitantly, this has stimulated ideas for reforms to medical and professional education to offer the requisite skills for delivering psychiatric care in resource-limited and emergency environments and how to coordinate with humanitarian relief efforts [5, 10, 92, 99]. Similarly, given the large-scale displacement generated by contemporary conflicts and diversifying patient populations, cultural competence is becoming an increasingly salient attribute in psychiatry, which could be better emphasized in training schemes [100].

Additional dynamic and pragmatic proposals have emerged arguing for the integration of geopsychiatry into core curricula to enhance attention to the macro-level determinants that are shaping the vulnerabilities of individuals and communities [10, 101]. Indeed, a recent global survey involving psychiatry faculty and residents found that only 4.2% reported high familiarity with geopsychiatry, yet 62.6% considered its incorporation into residency curricula as “very important” [101].

Ultimately, future clinical preparedness will likely require a combination of geopolitical awareness and local actions. Within an interconnected yet fracturing world, this ethos may well be necessary to build resilient systems capable of absorbing shocks, while protecting the dignity of those most in need of care and protection. The Austrian psychiatrist and Holocaust survivor, Viktor Frankl (1905–1997), once affirmed, “[w]hen we are no longer able to change a situation, we are challenged to change ourselves” [102]; as a psychiatric discipline and a global community, we would do well to heed this message.
